# Our chairman is very efficient: community participation in the delivery of primary health care in Ibadan, Southwest Nigeria

**DOI:** 10.11604/pamj.2017.27.258.12892

**Published:** 2017-08-07

**Authors:** Omowunmi Folake Iyanda, Oluwaseun Oladapo Akinyemi

**Affiliations:** 1 Association for Reproductive and Family Health, Ibadan, Nigeria; 2 Department of Health Policy and Management, College of Medicine, University of Ibadan, Ibadan, Nigeria

**Keywords:** Community participation, primary health care, spidergram, Nigeria

## Abstract

**Introduction:**

Community participation is rapidly being viewed as a requirement for the successful acceptance of health services; it integrates a complicated process which involves customs, beliefs, culture and power relations, not only structures and policies. Yet, there is a wide knowledge gap and changes favouring community participation in primary health care is still minimal. This study aims to assess the process indicators and other factors influencing community participation in the delivery of primary health care.

**Methods:**

This descriptive cross-sectional study using qualitative methods was conducted in Ibadan South East Local Government Area of Oyo State, Nigeria between July and September, 2015. The interview and Focus Group Discussion guides centred around five participation indicators of needs assessment, leadership, resource mobilization, organization and management was used to collect data. A total of 12 in-depth interviews and four FGDs were conducted among male and female respondents consisting PHC service providers and community members purposively selected from four wards of the LGA. Spidergrams were constructed to visualize the levels of community participation from respondents' opinions.

**Results:**

About 51.1% of the 45 respondents (with mean age 45.5 ± 8.09 years) were males. The respondents view community participation in the delivery of PHC in the LGA as being wide (open). Majority of the service users believe and agree that the level of community participation in their wards is about average while the service providers believed that participation was very high. However, respondents identified female representation, collaboration with pre-existing community structures, top-down and bottom-up approach to service delivery as factors affecting community participation in PHC delivery.

**Conclusion:**

This study provides a baseline data on community participation in the delivery of primary health care. Community participation is still an important principle in the delivery of primary health care and it guarantees the positive changes desired in the uptake and sustainability of primary health care programmes.

## Introduction

Primary health care is a grassroots approach towards universal and equitable health care for all as conceptualized by the Alma-Ata declaration of 1978 [1]. Several years later, its implementation is still below the optimum level in most Sub-Saharan African countries where access to health interventions remains a major problem for a large percentage of the populations [2]. Prior to the declaration, Nigeria had begun the implementation of Basic Health Services Scheme (BHSS) which was part of the Third National Development Plan from 1975-1980 [3, 4]. The BHSS however paid minimal attention to community participation, inter-sectoral collaboration and the use of appropriate technology; it was solely on the provision of health facilities and training of health workers [5]. The declaration at the Alma-Ata identified community participation, one of the pillars of primary health care; as the process by which individuals and families assume responsibility for the health and welfare of both themselves and the community thereby developing capacity to contribute to the growth of their community [1]. It is widely interpreted as the collective involvement of indigenous people in assessing their needs and strategizing to meet those needs [6]. For many decades, several advocacies have been made to support community participation in health as a strategy to improving health [7]. More than three decades ago, countries like Sri Lanka who had adopted the concept of primary health care showed obvious improvements in their health status and quality of life due to committed community participation among other reasons; in-spite of their under development as compared to the United Kingdom [8]. A study conducted in South Africa on tuberculosis treatment delivery and community participation in primary health care identified that community participation should be encouraged as better outcomes were recorded where communities participated [9]. Community participation is rapidly being viewed as a precondition for the successful acceptance of health services [10]. It integrates a complicated process which involves customs, belief, culture and power relations, not only structures and policies [11].

Community participation and community ownership is engendered by community mobilization to ensure the sustenance of health programs [12]. It is still crucial to the success of primary health care interventions as communities agree to this fact and desires greater involvement [2] which is likely to facilitate “needs-based and demand driving” provision of health services thereby promoting sustainability and ownership [13]. Recent studies have re-iterated the message that community participation is key to the delivery of health care [13], its importance cannot be over-emphasized. It is the combined participation of community members, their representatives and PHC personnel, to enhance their collective effort in eradicating health problems [14]. The PHC is specifically made to suit people at the community level. Gideon [15] submitted that “without the communities there would be no primary health care and without primary health care, communities will experience health problems”. A large and growing body of evidence exists that some forms of service delivery are improved when the communities they serve participate actively [16]. When the community members participate in defining problems, planning, implementation and evaluation of community resources, it gives them a sense of responsibility for their own health and also that of others [17]. Community participation was institutionalized in Nigeria through the creation of District Development Committee (DDC) and the Village/Community Development Committee (VDC) which are mandated to work in close proximity with local governments [17], to enhance the delivery and uptake of primary health care services. Despite this, reports have shown Nigeria's consistent underperformance in all health indices [18], however, some health care interventions such as nutritional programs, treatment of diarrhoea and Acute Respiratory Infections (ARI) symptoms have improved in the last few years; attributable to the incorporation of community participation and some of the principles of PHC for the strategic implementation of these programs [19]. This study provides a baseline data on community participation in the delivery of primary health care.

## Methods

**Study Area**: The study was conducted in Ibadan South East Local Government Area of Oyo state in the South-West geopolitical zone, Nigeria between July and September, 2015. The LGA was the headquarters of the defunct Ibadan Municipal Government and it is also known as the indigenous local government area. It has an estimated population of about 359,455 people of which about 22% are women of child bearing age [20, 21]. It covers an area of about 893 hectares. It has 12 wards and at least one PHC facility in each ward making a total of 16 PHC facilities out of which seven are actively functional.

**Study site and population**: Four wards (numbered from one to four; namely Beere/Mapo, Ojaba, Oranyan and Kobomoje) each with at least one PHC centre were purposively selected out of the 12 wards in the Local Government Area (LGA). The Medical Officer of Health (MoH) of Ibadan South East LGA (n=1), PHC service providers such as the local immunization officer, the disease notification and surveillance officer, the Health Educator for the LGA, the Chief matron of the LGA and the head of facility (n = 5), at least one Community Development Committee member per selected ward (n=6), male and female PHC service users who reside in the selected wards (n=33).

**Study design and sampling method**: The study was descriptive cross-sectional in nature using qualitative methods: Key Informant Interview (KII), In-depth Interview (IDI) and Focus Group Discussion (FGD). Purposive sampling was employed based on the inclusion criteria and willingness to participate; availability and ability to provide relevant information on the research questions [21]. Deliberate effort was made to recruit both male and female participants so as to explore the gender dimension of community participation. The FGD was conducted in the community, it involved male and female respondents and the discussions held separately. A moderator facilitated the discussions and ensured that every member of each group participated actively. A digital voice recorder was used for storing the data obtained from the interviews and FGDs and notes were also taken on paper to assist in the transcription process and for back up purposes. Four FGDs were conducted and each had 6-13 respondents.

**Figure 1:**
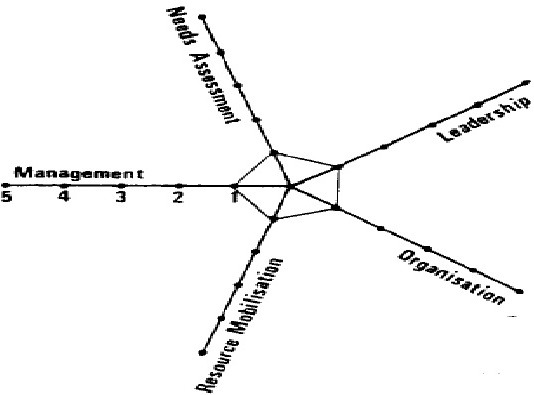
A Spidergram for assessing community participation (Rifkin, 1988)

**Spidergram**: The spidergram provides a simple, practical yet powerful way of illustrating the extent of community participation in important areas in a visual way as presented in Figure 1. The methodology identified five indicators from over 200 studies that can be used to measure, visualize and locate levels of community participation on a continuum, the indicators are; needs assessment, leadership, organization, resource mobilization and management [22]. The spidergram can also be used as an approach by health planners and program managers in assessing the reflection of changes in community participation showed via health outcomes and program impacts [23]. It has been used across several programs in several nations of the world [24] including Ghana where it was used in assessing participation in a community-based health planning and services program [10].

**Inclusion criteria**: Persons resident in the community for a minimum of 1 year, active members and participants of the Community Development Committee, Medical officer of health and PHC service providers all of who represent the stakeholders in PHC delivery and uptake in the LGA.

**Data collection and management**: An interview guide was developed for the key informant interview and the in-depth interviews and a focus group discussion guide was developed for the FGD. The interviews were semi-structured in accordance with a topic guide centered on the 5 participation indicators (needs assessment, resource mobilization, management, organization and leadership) and included open ended questions and “probe” questions. The interviews and discussions lasted for about 20-55 minutes. The FGD guide was translated and conducted in Yoruba language for easy comprehension and subsequently back-translated to English language to ensure that the original meanings were maintained. The focus group discussions with a minimum of six participants were conducted separately with male and female community members and the community development committee members to make the discussion homogenous as possible due to gender based opinion differences. The Medical Officer of Health (MoH) at the LGA served as the key informant while the in-depth interviews were conducted with the service providers Local Immunization Officer (LIO) and Disease Surveillance and Notification Officer (DSNO) > Disease Surveillance and Notification Officer (DSNO), the Health Educator at the LGA'> the Health Educator at the LGA, chief matron of the LGA and head of Oranyan PHC facility'> chief matron of the LGA and head of Oranyan PHC facility). Data obtained from the KIIs and IDIs were transcribed and the FGDs data was translated and transcribed in English language. The transcripts were imported into NVIVO version 8 software and analysed using the thematic framework approach to qualitative analysis [25]. It was a continuous process that commenced immediately after the first data was collected and continued throughout the research.

A thematic framework was developed for emerging themes at the end of each interview/discussions to perfect further interviews/discussions. The themes were indexed as they emerged and compared with themes of successive interviews/discussions until a sense of saturation was achieved and no new information could be obtained [26]. The spidergram of the extent of community participation in the wards and from the PHC service providers' perspective is constructed to visualize the present level of community participation [10, 22] as inferred from the results obtained. A score of 1-5 using the community participation assessment tool developed by Lehman, (1999) is placed on each process indicator located on a continuum on the spidergram to show how narrow or broad participation is in each of the selected wards. A score of 1 represents mobilization, 3-collaboration, 5-empowerment while 2 and 4 are intermediate values [23]. Ethical approval was obtained from the Oyo State Ministry of Health Research Ethics Review Committee before the commencement of the study. Permission to interview community members was sought from the Head of Local Government Administration, written informed consent authenticated with a thumb print/signature was obtained from each participant before the commencement of the interviews/ discussions and participants were assured of confidentiality. All interviews and discussions were conducted at a convenient time and place as chosen by the participants.

## Results

**Participants' characteristics**: A total of 45 persons participated in the interviews and focus group discussion sessions and each interview and discussion lasted for an average of 20-55 minutes. Majority of the participants were from the Yoruba tribe, which is the dominant tribe in the South west region of Nigeria. Table 1 shows the participants' characteristics while Table 2 provides the description of focus groups.

**Table 1:** Participants’ characteristics

Variable	n	%
**Gender**		
Female	22	48.9
Male	23	51.1
**Mean age(years±SD)**		
Female	46.8±7.56	
Male	44.5±8.62	
Overall	45.7±8.09	
**Tool**		
KII guide	1	2.2
IDI guide	5	11.2
FGD guide	39	86.7

**Table 2:** Description of focus groups

Code	Gender	Ward	Age group (years)	Number of participants
FGD1	Females	Ward 1	≥35	8
FGD2	Males	Ward 1	≥34	6
FGD3	Females	Ward 2	≥45	6
FGD4	Males	Ward 2	≥33	13

### Needs assessment

**Importance of needs assessment**: Respondents agree that community participation is important in community needs assessment and in the delivery of PHC and community members have been responsive to the PHC professionals in assessing some of their needs. According to a female community development committee member; community participation is very important in needs assessment as community members are the ones directly involved whenever a need arises' (IDI 2, female, W1). Another respondent opined that; Community participation cannot be overemphasized because PHC services is a healthcare service that is close to the grassroots and the community members have to be involved if not, they will not have a sense of belonging for that particular program (IDI 1, DSNO, male).

**Roles of community members in conducting needs assessment**: It was observed that community development committee members have a role to play in conducting needs assessment and PHC professionals have empowered some of them to play their roles in the wards in collaboration with international organizations such UNICEF, National Urban Reproductive Health Initiative (NURHI), World Health Organization (WHO) etc. They play active roles in mobilization and dissemination of information to their community. A respondent expressed how they empower the community to play their roles- “…the community people play very active roles especially in mobilization and advocacy. They support us in their own capacity and ensure that we get the necessary response from the community people, women and children inclusive” (KII, MoH).

**How the results of needs assessment are used**: The study showed that some results of needs assessment are used in planning PHC programs and it is dependent on the type of need assessed; It helps us to know the way forward, for example, there are certain communities in this LGA who didn't have health facilities; ward 4 and 9. How do they have a facility that they can call their own…we plan with them and ask questions like- who will give us the land? How do we build it? How much will it cost in terms of capital? … It all depends on the outcome of our plans with the community members to achieve our end results (IDI 3, LIO).

### Leadership

**Attitude of the leadership towards the introduction of PHC programs**: All the respondents had similar opinions that the leaders are positively responsive; a respondent opined that; “Our chairman is very efficient and he is highly interested in the health and welfare of the community. He has greatly helped our ward and we appreciate it” (FGD2, male 3). Although the health professionals emphasized that their responses were more favorable if they are given prior notice through community dialogue, yet they are very supportive as affirmed by the head of facility in ward 1; *for example, we had the family planning program introduced by an NGO, it was well accepted because this CDC served as a medium between us and the people, they did a lot in mobilizing their people for the program, they were available for the outreaches, they made their people available and sometimes they are involved in the planning and implementation of such programs* (IDI with the head of facility, W1).

**Representation of the leadership**: According to the respondents, the leadership represents different groups in the community, a male CDC opined that “The leadership represents the community, one of us is at the LGA council, some volunteers hold meetings with nurses at the PHC center to be privy of information regarding health, some of us attend the CDC meeting at Mapo too, (FGD2, male 2). Majority of the respondents were of the opinion that the leadership is active and takes initiative in community health activities although, female representation is not adequate and differs from ward to ward as observed during the discussions and interviews. In ward 1, the females were very active and they attended the discussions in large numbers, according to a male in ward 1; our women are very active in this ward and there is cooperation” (FGD2, male 4), unlike ward 2 where a female respondent was of the opinion that females are not adequately represented because most of our females are business women; they hardly have time for meetings except it is absolutely necessary. Just 2 females are part of the community development committee of 10 and we contribute when necessary, (FGD3, female 2). The health educator at the LGA buttressed this with her response; females are represented but not like the men, even in our community leaders meeting, we have just one female and in the SMC (Social Mobilization Committee) meeting, we have just two females in the midst of so many men, I noticed that females in this community are more concerned about their businesses, they rarely have time to participate, (IDI with the Health Educator).

**Criteria for choosing the leaders**: A number of criteria were highlighted by the respondents, majority were of the opinion that community members are solely responsible for selecting their leaders, that PHC professionals do not and cannot impose leaders on the community as opined by the medical officer of health who stated emphatically that “The community chose the leaders themselves, they do that within their various wards without any input from us, they just communicate the names of the leaders and members to us and we work with them” (KII, MoH). It was unanimously agreed that most of the decisions by the leadership has resulted in health improvement for the poor and elites.

**Resource mobilization**: Interviews of service providers and discussions with community members and service users revealed that resource mobilization and provision is dependent on the type of resources needed; whether human, material or financial resources.

**Resource mobilizers and influencers**: According to the respondents, resources could be solely provided or influenced by; the community members, philanthropists, the government at the federal, state or local level and international organizations and several approaches are used in the mobilization of resources. There is a continuing contribution of indigenous resources but the community has minimal control of resources which is dependent on the type of resources raised. In a service provider's opinion: The development partners are the major people who make resources available for PHC. For instance, the NHIS provides free health services to children 0-5 years and pregnant women. Community members are involved in resource mobilization, “some sell their buildings or donate it for PHC”, (IDI 1, DSNO). It was also discovered that various already existing community groups influence mobilization of resources, although a female respondent had a contrary opinion that “there is no group that influence mobilization of resources for PHC for now, although I know the group will be set up when the need arises” (Interview with a Female in ward 4).

**Organization**: There is integration and no conflicts between the pre-existing community structures (such as traditional birth attendants (TBAs), voluntary health workers (VHWs), etc.) and Primary Health Care from the findings of this study. The study revealed that the TBAs are especially integrated with PHC, they even hold their meetings regularly in the major health center in the LGA, although there was a breach in communication in the past and there are measures presently in place to ensure smooth integration according to the MOH. A community participant re-iterated this during a discussion, according to him; “there is no conflict at all. I am a TBA, whenever we take care of pregnant women and discover there are complications, we refer them to the center” (FGD4, male 5). Existing community structures have been absorbed and co-operates actively in PHC activities, this is to ensure their full participation as opinionated by a participant who said they have been trained, they have been identified and they are working. When we are implementing certain programs, they will call the chairman of the TBAs and ask for about four of them who will be used as volunteers. When we train them, we let them know their boundaries (IDI 2). Majority of the trained VHWs however were of the opinion that they are not properly compensated by the PHC professionals for the roles they play in mobilization and advocacy; a female participant said “they tell us to go out for advocacy, yet they don't take good care of us. The last time my daughter went there, she was not well treated, yet, they tell us to sensitize our people that health care is free, still they make us pay heavily to assess care” (FGD1, female 5).

**Management**: This study revealed that community members through their representatives in the CDC are involved in decision making with respect to PHC and majority agreed that their representatives have managed programs effectively in their capacity, although the extent of their management roles is dependent on that given by the PHC professionals.

**Roles of community members in the management of PHC programs**: Their roles vary from time to time, “some are given the responsibility of sensitization and awareness, resource pooling and they are used as facility assistants” (IDI with the head of Ward1 facility). A participant stated clearly that PHC has been well managed in our ward, the PHC officials don't take decisions without our consent, for example, when they want to organize child health week, they enlighten us and give us handbills to share from house to house and to people in religious centers for awareness. They do that from ward to ward. They go to places where there are quite a number of children to also enlighten them so they can bring their children for immunization. PHC is well managed in our community here. A PHC worker emphasized that “No program can succeed without the involvement of the community members in the planning and implementation” (IDI 1, DSNO).

**Respondents' view on how PHC has fared in the wards, LGA and in Nigeria**: All respondents believe that PHC has fared well in their wards, LGA and in Nigeria as a whole. According to a respondent, “Since the Alma Ata declaration, in my opinion, PHC has fared very well in Nigeria. The introduction of PHC has seen the breaking of a new day for health care in Nigeria. I must say we still have a lot to do, but so far, we cannot ignore the fact that we are better than we were before the introduction of PHC in Nigeria” (KII, MoH). Some were of the opinion that the PHC professionals and the government are doing well with respect to Primary Health Care implementation in Nigeria, although others had contrary opinions particularly the professionals who have a detailed understanding of what PHC really is and eluded it to the constraints so far experienced with its implementation.

**Figure 2:**
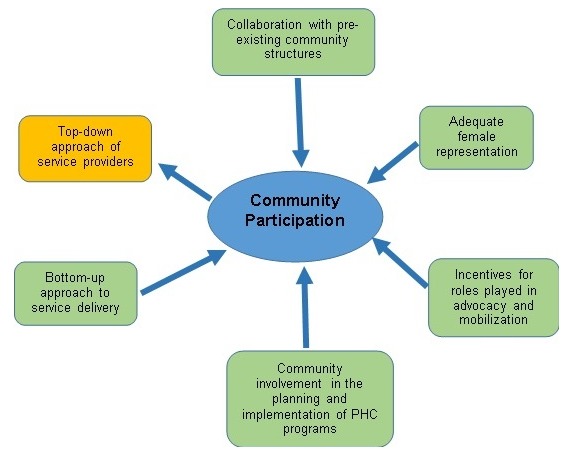
Factors influencing community participation

**Figure 3:**
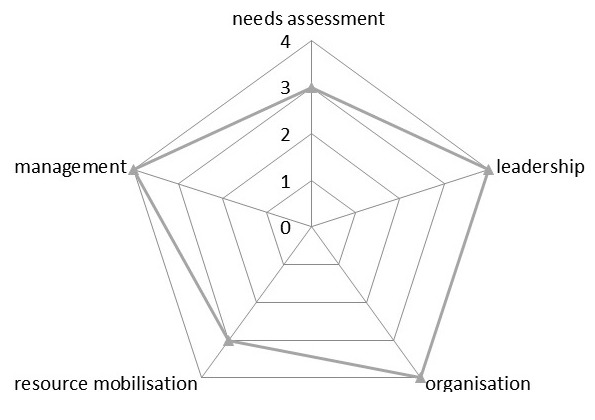
Spidergram showing community members' view of their participation

**Figure 4:**
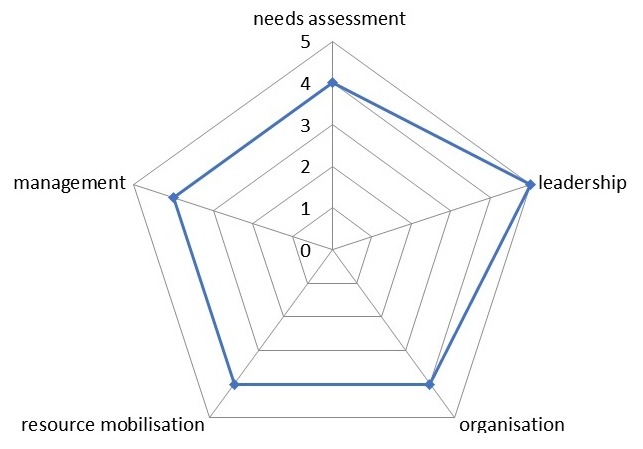
Spidergram presenting PHC service providers' view of community participation

**Figure 5:**
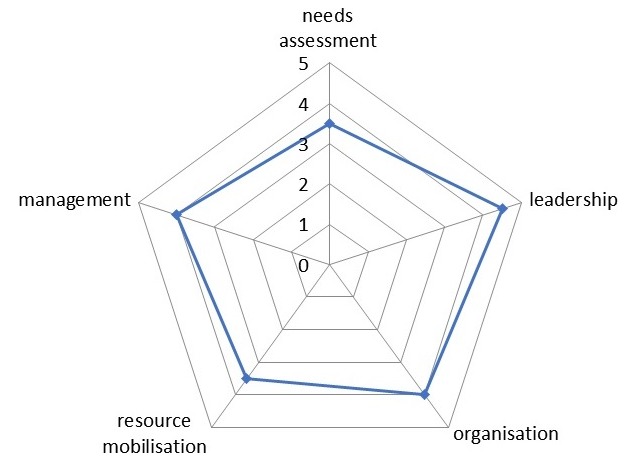
Spidergram displaying the collective opinion of all respondents on the level of community participation in the LGA

**Factors affecting community participation in the delivery of Primary Health Care**: In the course of data collection, several other factors were identified as either favoring or hindering community participation in the delivery of Primary Health Care in the LGA. The principal factor identified as a hindrance to community participation was top-down approach by some service providers while involving community members by keeping them abreast of the planning and implementation of Primary Health Care programs, adequate female representation and giving community members and leaders incentives were necessary factors that facilitated community participation. Figure 2 illustrates factors influencing community participation as identified by participants. From the findings of this study, PHC service providers and community members have a slightly different opinion on the current level of community participation for PHC delivery in the LGA as visualized on the spidergram presented in Figure 3, Figure 4, Figure 5. A community participation assessment tool adapted from Karen (1999) was used to rank the process indicators on a spidergram. Each indicator is presented on its own as a continuum and linked to the other four. One end represents narrow participation (mobilization) and the other represents wider participation (empowerment). Figure 3 shows that the community view their participation in PHC programs as generally being wide; however, their involvement in needs assessment and resource mobilization is intermediate. The PHC service providers were of slightly differing opinions compared to the community members' view, in resource mobilization, management and needs assessment as shown in Figure 4. Figure 5 shows the collective opinion of both service providers and community members revealing wide participation in the empowerment end.

## Discussion

This study aims to assess community participation, as a major principle in the delivery of Primary Health Care and it uses the Spidergram as a methodological tool to visualize participation levels. All the study participants admitted that PHC has fared well in Nigeria since the Alma-Ata declaration and the importance of community participation in its delivery cannot be over emphasized. This agrees with Makaula and colleagues' [2] view that community participation is crucial to the success of PHC. Respondents were however of the opinion that communities have different needs and participation is situation specific as postulated in a systematic review of the literature for evidence on health facility committees in low- and middle-income countries by McCoy et al [7]. This research work found that community participation could take several forms; via mobilization, planning, needs assessment, advocacy, resource mobilization etc. but no matter how narrow or wide it is, there is always some form of community participation. This agrees with the findings of Draper [23] et al in developing indicators for community participation. The spidergram shows the participation level in the wards; it can also be used to monitor community participation changes in specific PHC programs, thus agreeing with Draper et al [23] recommendations. The spidergrams constructed from the findings of this study reveals that the overall community participation in the delivery of PHC is wide as it situates clearly towards the empowerment end of the participation continuum. However, it further reveals that needs assessment and resource mobilization as indicators of participation needs to be improved upon to further widen the participation level. This can be used as a baseline comparative tool for community participation assessment in future community based health programs in Ibadan South East LGA and invariably in Ibadan metropolis. It can also be used to link health outcomes in already implemented programs.

Community's contribution of indigenous resources and integration of PHC services with the pre-existing community structures favoured community participation in the delivery of Primary Health Care, thus agreeing with literature that described that the resources of community health committees are significant factors in community participation for health [6, 11]. This study revealed that community members through their representatives supported the delivery of PHC by contributing lands/ buildings; the results from the survey of PHC facilities by Gupta et al [27] which showed that community health committees were the major source of support for building maintenance in majority of the selected PHC facilities agrees with this finding. Although, some community members belief that their requirements to mobilizing resources for PHC services is a way of diverting the responsibilities of the government; this aligns with the findings of McCoy et al [7] findings that governments may use community participation in health to divert their responsibilities to communities. In some wards, it was observed that female participation was very minimal; consistent with findings from the assessment of participation in a community-based health planning and services program in Ghana [10]. Male dominance, from the results of this study has a negligible effect on whether the community members will participate or not, this is however inconsistent with the findings of Baatiema and colleagues [10] and Sepheri and Pettigrew [28] that male dominance hindered community participation and prevented community health committees from effectively representing the interest of the community as a whole thereby preventing total community participation in PHC delivery. A number of factors were inferred from respondents' discussions and interviews as favoring or hindering community participation in Primary Health Care delivery. The general opinion of respondents, particularly community members is that inadequate mobilization and advocacy for programs is a major factor hindering their participation; this is corroborated by Alenoghena et al view [29]. The results of this study revealed the desire of community members for monetary incentives; especially those who participate in mobilization and advocacy, some even noted their desire to be on a regular payroll of the PHC professionals which agrees with the findings of Kironde and Klaasen [9] on the desire of volunteers in the community for remuneration in developing countries as a major hindrance to community participation. This is also corroborated by the findings of McCoy et al [7] that communities are expected to participate through their health committees on an unpaid basis.

The present economic state of the country and the literacy level of the community members however might be a contributing factor to this finding. This study also revealed that PHC staff-responsiveness and accountability was substantial where communities participated in PHC delivery; this is corroborated by findings of Gupta et al [27]. Community members view Primary Health Care programs in which they participate as beneficial to them and they sometimes completely ignore the uptake of services in which they feel they have minimal participation; this agrees with the submission of Preston et al [30] that community participation can increase the uptake of services and yield favorable health outcomes. The opinions of PHC professionals and those of community members (CDC members inclusive) differed slightly from each other in a few instances which may be due to reporting bias as postulated by Baatiema and colleagues [10]. This study also revealed that community participation is a complex process as opined by Mosquera [11] et al (2001) that involves customs, belief, culture and power relations, not just about structures and policies. The proper functioning of the CHC/CDC was discovered to be influenced by local political structure; this corresponds with the findings of Mubyazi et al [31] on the poor functioning of village health committees in Tanzania due to political divides and Uzochukwu et al [32] findings on community members' refusal to participate in health activities as a result of the opposition to the leadership of the committee among other reasons. Some community members were also of the opinion that poor quality services were rendered in their health centre, therefore their reason for refusal to support PHC service delivery; this supports the findings of Mubyazi and Hutton [33] that weak district and primary health care systems undermined participation. Another key finding in this study is that communities are willing to participate fully when they view their PHC providers as being humble and approachable; this is in agreement with Sohani’ [34] experience with dispensary health committees effectiveness in Columbia where younger nurses enhanced community participation in health because they were unlikely to have high egos.

**Limitations**: The results obtained in this study are based on the subjective perspective of a few people in the population and therefore might not be generalizable to the entire population. In addition, saturation of data [35] might not be guaranteed due to the limited number of interviews and focus group discussions. However, this study provides useful preliminary data on the role of community participation in the implementation of PHC in Ibadan, Southwest Nigeria.

## Conclusion

Findings from this qualitative research provides evidence that community participation is still an important principle in the delivery of Primary Health Care. The primary health care system in the local government area of study can be said to be people-centred as the roles, capabilities (in terms of resource mobilization, leadership and advocacy), needs and preferences of community members were given priority in the daily operation of the health system. However, it is still pertinent for all stakeholders (particularly the government at the federal, state and local level) to critically track and ensure that community participation is encouraged in the delivery of primary health care. This will possibly guarantee the positive changes desired in the uptake and sustainability of primary health care programs. Community participation in primary health care delivery should be strengthened and not ignored as a pillar of primary health care, the professionals should ensure that community members fully participate in the areas of planning and implementation of programs. Community members and their representatives should continually support their primary health care service providers especially with respect to community mobilization and advocacy for primary health care programs. Agencies responsible for primary health care in Nigeria should visit communities to evaluate their participation in primary health care delivery from time to time. The results obtained in this evaluation should be used in modifying policies to suit the delivery of primary health care in order to ensure that community members as target population of programs are really benefiting from programs.

### What is known about this topic

- Community participation is an important concept in the implementation of primary health care and the realisation of universal health care;
- Community participation is a precondition for the successful acceptance of health services in communities.

### What this study adds

- Community development committee members have a role to play in conducting needs assessment, mobilization and dissemination of information within their communities;
- Factors enhancing community participation in the delivery of primary health care include adequate female representation, incentives for community members for roles played in advocacy and mobilization, community involvement in planning and implementation, bottom-up approach to service delivery and collaboration with existing community structures;
- Top-down approach to primary health care by some service providers impedes community participation.

## Competing interests

The authors declare no competing interest.
